# Assessment of Cardiorespiratory Fitness in Individuals With Type 2 Diabetes Mellitus: Protocol for a Systematic Review and Meta-Analysis

**DOI:** 10.2196/91185

**Published:** 2026-09-22

**Authors:** Gajanan Patil, Nazli Khatib, Alka Rawekar, Adwait Godse, Laxmikant Umate

**Affiliations:** 1 Department of Physiology Jawaharlal Nehru Medical College Datta Meghe Institute of Higher Education and Research Wardha, Maharashtra India; 2 Department of Physiology Shri Vithalrao Joshi Charities Trust / B.K.L. Walawalkar Rural Medical College Ratnagiri, Maharashtra India; 3 Datta Meghe Institute of Higher Education and Research Wardha, Maharashtra India

**Keywords:** type 2 diabetes mellitus, cardiorespiratory fitness, pulmonary function, 6-minute walk test, heart rate variability

## Abstract

**Background:**

Reduced cardiorespiratory fitness (CRF) is a well-recognized but underaddressed complication of type 2 diabetes mellitus (T2DM), contributing substantially to cardiovascular morbidity, mortality, and reduced quality of life. Evidence related to pulmonary function, functional exercise capacity, and autonomic dysfunction in T2DM remains fragmented and methodologically heterogeneous.

**Objective:**

This protocol describes a systematic review and meta-analysis that aims to synthesize evidence on CRF in adults with T2DM, evaluate associated clinical and demographic determinants, and examine relationships with glycemic control and diabetes-related outcomes.

**Methods:**

The protocol is registered with PROSPERO (CRD420251009300) and follows PRISMA-P (Preferred Reporting Items for Systematic Review and Meta-Analyses Protocols) guidelines. A comprehensive search of PubMed, Cochrane Library, Web of Science, ClinicalTrials.gov, World Health Organization’s International Clinical Trials Registry Platform, and gray literature will be conducted for studies published between 2013 and 2024. Randomized controlled trials, cohort studies, and cross-sectional and case-control studies involving adults with T2DM will be included. Primary outcomes include pulmonary function test parameters, 6-minute walk test distance, and heart rate variability indexes. Two reviewers will independently screen studies, extract data, and assess risk of bias using the Revised Cochrane Risk-of-Bias Tool for Randomized Trials (RoB 2), Newcastle-Ottawa Scale, and the JBI critical appraisal tools, as appropriate. Meta-analyses will be performed using random-effects models when sufficient homogeneity exists. The GRADE (Grading of Recommendations Assessment, Development and Evaluation) framework will be used to assess certainty of evidence.

**Results:**

The study was approved by the Doctoral Research Committee on December 19, 2024, and the Standing Scrutiny Committee and Institutional Ethical Committee on October 1, 2025. Data collection began after approval in December 2024, and the meta-analysis was completed in April 2026. The results of the meta-analysis will be published in August 2026.

**Conclusions:**

The findings will support evidence-based clinical assessment and guide future research on integrative cardiopulmonary and autonomic evaluation in T2DM.

**Trial Registration:**

PROSPERO CRD420251009300; https://www.crd.york.ac.uk/PROSPERO/view/CRD420251009300

**International Registered Report Identifier (IRRID):**

DERR1-10.2196/91185

## Introduction

### Background

Diabetes mellitus (DM) is a long-term metabolic disorder marked by persistently elevated blood glucose levels, resulting from inadequate insulin production, impaired insulin effectiveness, or a combination of these factors [1]. Globally, diabetes mellitus poses a substantial threat to public well-being, significantly impacting illness rates and death tolls, thus presenting a formidable challenge for health care systems worldwide [2]. Estimates from 2017 indicated more than 425 million individuals were affected worldwide. India, bearing a substantial burden, reported 73 million adults diagnosed with type 2 diabetes mellitus (T2DM), with projections suggesting a dramatic rise to 134 million by 2045 [3]. This escalating prevalence, particularly acute in low-income countries, poses challenges for health care infrastructures often unprepared for the mounting demand [4]. T2DM, frequently termed a lifestyle disease, imposes considerable social, epidemiological, and economic costs. The management of this issue requires substantial health care resources and reduces productivity at both the individual and societal levels, thereby reinforcing its designation as a critical concern [5-7].

### Pathophysiology and Complications

T2DM disease progression initiates at the cellular level, where chronic glucose elevation activates a series of metabolic disruptions [5]. These biochemical modifications translate into the principal clinical manifestations of type 2 diabetes, which develop through damage to both microscopic blood vessels (microangiopathy) and major arteries (macroangiopathy) across various organ systems [6-8]. At the molecular level, persistent high blood sugar triggers the nonenzymatic attachment of glucose to proteins, including collagen and elastin [3,9]. This biochemical modification causes basement membranes to become thicker and promotes microvascular complications [10-12]. Small vessel complications primarily target the retina, kidneys, and peripheral nerves, while large vessel disease accelerates arterial plaque formation and significantly elevates cardiovascular disease risk [4,9-12]. The incidence of cardiovascular disease is notably more prevalent in individuals diagnosed with T2DM compared to those without this condition [13]. Vascular damage induced by diabetes is associated with a two- to fourfold increase in the risk of coronary heart disease and stroke, alongside a 2- to 8-fold elevation in heart failure incidence [14]. The heightened cardiovascular disease prevalence results in about 80% of mortality cases among patients with T2DM, making heart and vascular conditions the most common cause of death within this demographic [15-17]. The fundamental pathways responsible for these cardiovascular issues involve atherosclerotic plaque formation with thrombosis, diminished arterial elasticity, dysfunction of the microvasculature, and impaired cardiac autonomic nervous system activity [18,19]. Research evidence has solidly established the connection between type 2 diabetes and cardiac autonomic nervous system dysfunction, illustrating that autonomic abnormalities develop before clinical cardiovascular manifestations and serve as independent determinants of cardiac morbidity and sudden mortality [4,20-22]. Long-term increased blood glucose levels in patients with diabetes are associated with widespread organ damage and impairment across various body systems, including vascular networks, neural pathways, muscular tissue, ocular structures, renal function, and cardiac health [8].

### Benefits of Physical Activity

Regular physical exercise is beneficial for patients with T2DM, providing various health improvements including diminished risk of diabetic complications, better blood sugar regulation, enhanced insulin sensitivity, favorable lipid modifications, blood pressure reduction, effective weight maintenance, and improved life quality [2,9,16,23-26]. People with type 2 diabetes often lead less active lives, which gradually weakens their ability to exercise and perform daily tasks. Cardiorespiratory fitness (CRF)—the body's combined cardiovascular, respiratory, and muscular efficiency in delivering and using oxygen during activity—stands out as a key marker of functional health. It also powerfully predicts risks of heart disease, illness, and death [27,28]. Global diabetes authorities such as the American Diabetes Association and European Association for the Study of Diabetes, along with international clinical standards, endorse regular exercise as an essential element in diabetes treatment due to its wide-ranging physiological benefits [29]. Individuals with diabetes indicate reduced exercise capacity, early physical health decline, and that it carries substantial prognostic implications. The pathophysiological mechanisms underlying reduced exercise capacity involve interrelated myocardiogenic, myogenic, vasculogenic, and neurogenic factors [4,21].

### Assessment of CRF

Assessing cardiopulmonary fitness in individuals with T2DM is crucial as it provides insights into overall health status, helps identify early signs of cardiovascular and pulmonary complications, and serves as a powerful prognostic marker [21,22,24,27,30,31]. Reduced cardiovascular fitness is strongly associated with an increased burden of disease and elevated mortality rates among individuals with type 2 diabetes. Notably, each improvement of one metabolic equivalent (MET) in fitness is linked to a 10%-25% reduction in the risk of cardiac-related mortality [30-33]. This assessment becomes even more important considering that many diabetic complications remain asymptomatic until advanced stages, highlighting the need for objective functional evaluation [27,34,35].

Widely used CRF assessment methods include pulmonary function tests (PFTs), 6-minute walk test (6MWT), and heart rate variability (HRV). These assessment tools provide complementary information about different aspects of cardiopulmonary health and can be integrated into routine clinical care to guide management strategies and monitor disease progression or treatment response [4,7,22,29,36].

### Current Knowledge of CRF in T2DM

Cardiovascular endurance represents an essential health parameter for individuals with T2DM and has shown greater prognostic accuracy for death risk than standard cardiovascular risk variables [9,25]. Research has convincingly demonstrated that reduced CRF in T2DM is associated with increased cardiovascular morbidity and mortality, with diabetic individuals typically showing 20%-30% lower peak oxygen consumption compared to age-matched controls [2,15,22,34]. Inadequate blood sugar management, reflected by high hemoglobin A_1c_ (HbA_1c_) levels, is associated with diminished cardiovascular fitness capacity [18,20,32]. The causality and directionality of this relationship remain subjects of debate. Some studies suggest that hyperglycemia directly impairs mitochondrial function and skeletal muscle metabolism, while others propose that lower CRF predisposes to insulin resistance and hyperglycemia [14,22,28].

Exercise capacity, evaluated using the 6MWT, is often reduced in these individuals due to cardiovascular, respiratory, and muscular limitations [5,26,29,37-39]. The prevalence of reduced HRV increases with diabetes duration, with some studies reporting autonomic dysfunction in up to 65% of long-standing T2DM cases [40-44]. Structured exercise programs improve CRF in T2DM, alongside better glycemic control and enhanced quality of life [17,25,45]. The evidence suggests that both aerobic and resistance training modalities offer benefits when appropriately prescribed and progressively implemented [29,32,33,37].

### Knowledge Gaps and Research Needs

Despite extensive research, important knowledge gaps persist in our understanding of cardiopulmonary fitness in T2DM. Most studies focus on single measures of CRF, such as maximal oxygen uptake (VO_2_ max) or 6MWT distance, with limited research simultaneously assessing pulmonary function, exercise capacity, and autonomic function in T2DM populations. This compartmentalized approach fails to capture the integrated nature of cardiopulmonary dysfunction in diabetes and may miss important interactions between different physiological systems [23,36,41].

A recent comprehensive review by Macedo et al [46] concluded that T2DM is associated with lower CRF, evidenced by reduced VO₂ max and shorter 6MWT distances across multiple studies. However, the study identified important limitations in the existing literature, such as underrepresentation of older adults, poor methodological quality, and high heterogeneity in outcomes and assessment protocols [46].

Despite growing evidence on pulmonary impairment, reduced functional capacity, and autonomic dysfunction in T2DM, several knowledge gaps persist. Findings across studies are inconsistent, often limited by cross-sectional designs, varied methodologies, and lack of standardization. The underlying mechanisms and the impact of interventions like exercise or glycemic control on pulmonary function, 6MWT performance, and HRV remain unclear. Additionally, limited subgroup analyses and absence of longitudinal data highlight the need for more comprehensive and targeted research in this area.

A systematic review is essential to synthesize the fragmented and often inconsistent evidence on pulmonary function, functional capacity (6MWT), and HRV in T2DM. Existing studies vary in methodology and findings, limiting clear conclusions about the extent of impairment, its relationship with glycemic control, and its clinical implications. A comprehensive review would help quantify these associations, evaluate test validity, and guide clinical practice and future research on early detection and management of cardiopulmonary and autonomic complications in T2DM.

Addressing these gaps by including autonomic function assessments (HRV) alongside traditional cardiopulmonary measures may enable clinicians to interpret cardiopulmonary tests more effectively, enhance risk stratification in diverse diabetic populations, and lead to better-targeted interventions tailored to specific patterns of dysfunction [23,36,41,46].

### Significance and Potential Impact

This study is poised to offer several noteworthy contributions. Patients stand to gain from enhanced risk assessment, and the implementation of more personalized management strategies could result in improved health outcomes and quality of life [22]. Clinicians may benefit from advanced tools for patient evaluation, enabling more informed decision-making in treatment planning [7]. Researchers are likely to obtain new insights and directions for future investigations into interventions designed to enhance cardiopulmonary fitness in individuals with T2DM [28]. Policymakers will have access to new data that can inform public health strategies and guide resource allocation for diabetes management. Health care systems may experience more efficient and effective management of patients with T2DM, potentially mitigating long-term complications and reducing health care costs.

### Objectives

This study aims to (1) systematically evaluate the CRF in patients with T2DM compared to healthy individuals, (2) identify factors (age, sex, diabetes duration, BMI, glycemic control [HbA_1c_], and physical activity levels) associated with CRF in patients with T2DM, (3) determine the association between the CRF and glycemic control (eg, HbA_1c_ levels) in patients with T2DM, (4) investigate the relationship between CRF and all-cause mortality or diabetes-related complications (eg, neuropathy and retinopathy) in patients with T2DM, and (5) provide evidence-based recommendations for improving CRF in patients with T2DM.

## Methods

### Study Design

This systematic review and meta-analysis will adhere to the PRISMA (Preferred Reporting Items for Systematic Reviews and Meta-Analyses) reporting standards. The review protocol is registered with PROSPERO (CRD420251009300), the International Prospective Register of Systematic Reviews.

### Eligibility Criteria

The participant eligibility criteria are presented in Table 1.

**Table 1:** Participant eligibility criteria.

	Inclusion criteria	Exclusion criteria
Study	Cohort studies Cross-sectional studies Randomized controlled trials Nonrandomized controlled trials	Prestudies and poststudies without a control group Case reports, case series, and qualitative studies Studies with inadequate reporting of methodology Duplicate publications of the same study Animal studies
Population	Adults aged 30-75 years diagnosed with type 2 diabetes mellitus	Adults with type 1 diabetes mellitus History of smoking and self-reported proof of any pulmonary and cardiac disease Cardiovascular diseases (eg, heart failure and recent MI^a^) End-stage renal disease, etc
Outcomes assessed: cardiorespiratory fitness measurement methods	Spirometry—for lung function test 6-minute walk test—functional capacity measurement Heart rate variability—autonomic function measurement All-cause mortality: diabetes-related complications (eg, neuropathy and retinopathy)	—^b^
Other relevant details	Source of funding: potential conflict of interest statements from all included studies: no Any other relevant details, if any	Non-English language

^a^MI: myocardial infarction.

^b^Not applicable.

### Information Sources and Search Strategy

#### Electronic Searches

A thorough search of electronic databases, trial registries, and gray literature will be conducted. Sources include MEDLINE (via PubMed); Cochrane Central Register of Controlled Trials (Cochrane Library); SPORTDiscus; Google Scholar; clinical trial registries; gray literature (OpenGrey and ProQuest Dissertations); hand-searching of key journals; and direct outreach to field experts, researchers, and organizations. The search covered studies published from January 2014 to December 2024.

#### The Search Strategy

Searches will combine MeSH terms, keywords, and related entries such as “Cardiopulmonary Fitness,” “Pulmonary Function,” “6-Minute Walk,” and “Heart Rate Variability,” paired with “Type 2 Diabetes Mellitus.”

### Screening and Selection of Studies

The results of the search will be exported as a CSV file and then imported into Rayyan.ai screening software. Each study will be initially screened based on the title and abstract. Relevant studies identified during the initial screening phase will then be assessed based on the full text.

### Data Extraction

Two independent reviewers (GP and AG) will extract data from eligible studies using a pretested, standardized Microsoft Excel form. The following data will be extracted: study characteristics; geographic factors such as country and region; study design, type, and setting; and the unit of analysis. Any disagreements between the 2 reviewers during data extraction will be resolved through discussion to reach consensus. If consensus cannot be reached, 2 additional reviewers (AR and NK) will be consulted to make the final decision.

### Participant Details

Participant characteristics include the number of participants, mean age, male-to-female ratio, duration of diabetes, mean HbA_1c_ levels, physical activity levels, and BMI.

### Study Selection

After database searches and screening, studies that meet the predefined eligibility criteria will be included in the qualitative synthesis. The final selection will include case-control studies, analytical cross-sectional studies, and prospective cohort studies, representing a diverse array of observational designs that evaluate the outcomes of interest. The study selection process is depicted in the PRISMA flow diagram (Figure 1).

**Figure 1:**
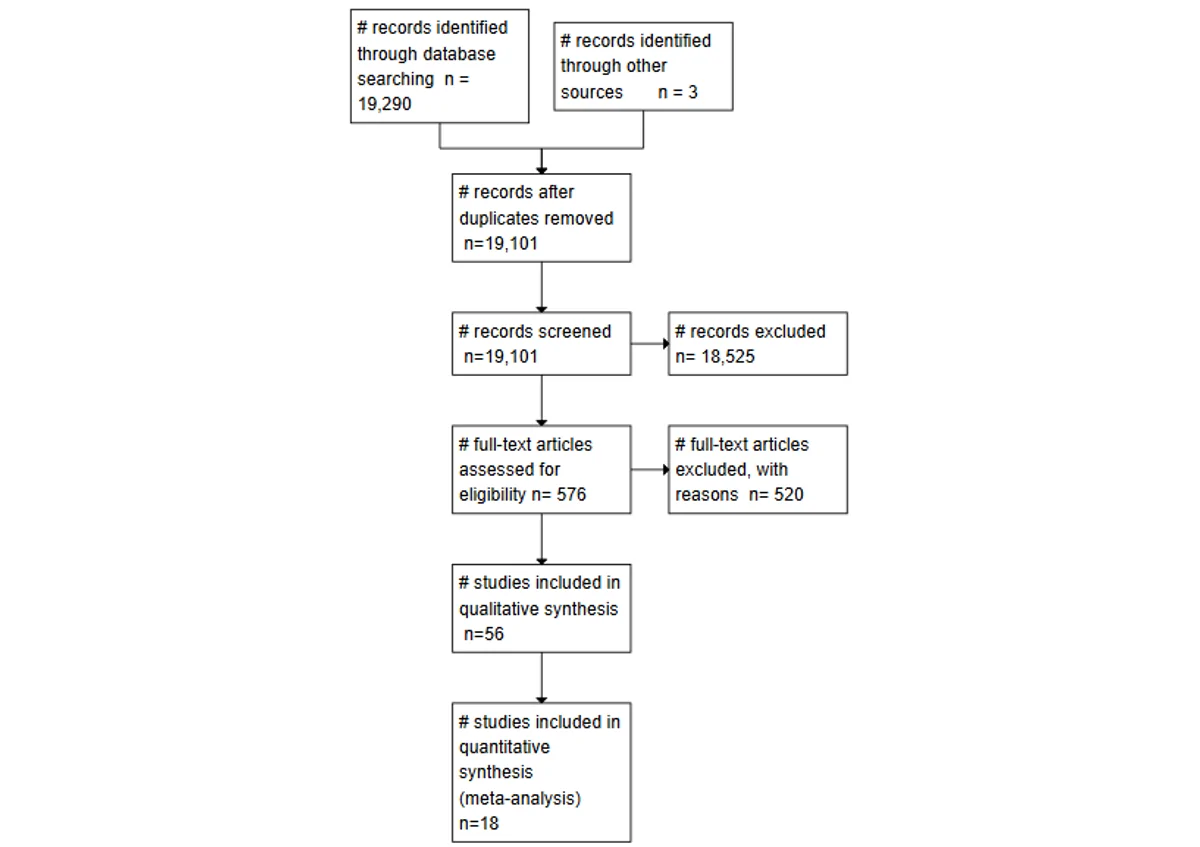
PRISMA (Preferred Reporting Items for Systematic Review and Meta-Analyses) flow diagram.

### Study Characteristics

The included studies will be descriptively summarized in a table presenting their key features. Common elements will include study design (eg, case-control and cohort), population demographics, interventions or exposures, outcomes measured, and geographic settings. This will provide an overview of the scope and heterogeneity of the available evidence.

### Risk of Bias Assessment

Risk of bias will be assessed using the Revised Cochrane Risk-of-Bias Tool for Randomized Trials (RoB 2) for randomized trials, Newcastle-Ottawa Scale for cohort studies, and JBI tools for case-control and cross-sectional studies.

### Outcomes Assessed

The outcomes included CRF, assessed using spirometry for lung function, the 6MWT for functional capacity, and HRV for autonomic function. Additional outcomes encompassed all-cause mortality and diabetes-related complications, such as neuropathy and retinopathy. Additional pertinent details, such as funding sources and potential conflict of interest statements from all included studies, will be thoroughly addressed. If any data are missing, the authors will be contacted via email to request the necessary information. The study selection process will be meticulously documented using a PRISMA flowchart. This chart will illustrate the number of research papers evaluated against the inclusion criteria and those ultimately incorporated into the systematic review, along with a comprehensive outline of the screening process that clarifies the reasons for excluding studies.

### Synthesis of Results

Meta-analysis will be conducted using STATA software (version 20; StataCorp) to perform the quantitative evaluation of the studies selected for inclusion. Meta-analytical techniques will be applied when adequate study homogeneity is present. For continuous variables (including PFT, HRV, walking distance, and VO_2_ max), data will be reported as mean differences (MDs) or standardized mean differences (SMDs) with corresponding 95% CIs comparing participants with and without T2DM. A random-effects model will be used for data pooling to accommodate interstudy heterogeneity.

### Subgroup and Sensitivity Analyses

Subgroup analysis will be used when the number of available studies is insufficient for meta-regression (<10 studies per subgroup). Subgroup analyses will explore age, sex, BMI, glycemic control, and diabetes duration. Sensitivity analyses will assess robustness by excluding high–risk of bias studies.

### Publication Bias

Funnel plots and Egger and Begg tests will be used to assess publication bias when sufficient studies are available.

### Grading the Quality of Evidence

The quality of evidence will be evaluated using the GRADE (Grading of Recommendations Assessment, Development and Evaluation) approach. This assessment will account for risk of bias, inconsistency of results, indirectness of evidence, imprecision, and publication bias.

### Ethical Considerations

This study has been approved by the institutional ethical committee of Datta Meghe Institute of Higher Education and Research (deemed to be exempt; IEC/2025/548; dated October 1, 2025).

## Results

The study was approved by the Doctoral Research Committee on December 19, 2024. The Standing Scrutiny Committee and Institutional Ethical Committee approved the study on October 1, 2025. After approval, data collection began in December 2024. The meta-analysis was completed in April 2026. The results of the meta-analysis will be published in August 2026.

## Discussion

### Rationale and Significance of the Review

T2DM is associated with a wide array of cardiovascular, pulmonary, and autonomic dysfunctions, collectively diminishing CRF and overall functional capacity. Increasingly, assessments such as PFTs, the 6MWT, HRV, and various cardiopulmonary parameters are being used to evaluate these deficits. However, the existing body of evidence remains fragmented, displaying considerable variability in study designs, participant characteristics, outcome measures, and analytical methods.

This systematic review and meta-analysis aim to synthesize available evidence on the association between CRF parameters and T2DM, thereby addressing a critical gap in the literature. By integrating data from observational and interventional studies, this review seeks to provide a comprehensive and methodologically rigorous summary of current evidence, which may help clarify the extent and clinical relevance of cardiopulmonary impairment in individuals with T2DM.

### Anticipated Contributions to Clinical Practice and Research

The findings of this review are expected to inform clinicians, physiotherapists, and researchers about the potential role of CRF assessments as adjunctive tools in the routine evaluation of individuals with T2DM. Identifying consistent associations between reduced CRF and glycemic or metabolic dysregulation may support the integration of functional assessments, such as the 6MWT, HRV analysis, and spirometric indices, into diabetes care beyond traditional biochemical markers.

From a research perspective, the review is expected to highlight areas of consistency as well as gaps in the existing literature, particularly with respect to methodological quality, confounder adjustment, and outcome standardization. This may guide future longitudinal studies and randomized controlled trials aimed at improving cardiopulmonary outcomes through targeted exercise or lifestyle interventions in T2DM populations.

### Strengths of the Review

A key strength of this review lies in its comprehensive and systematic approach, including a broad search strategy across multiple electronic databases and gray literature sources. The use of standardized methodological frameworks, such as PRISMA-P (Preferred Reporting Items for Systematic Review and Meta-Analysis Protocols) guidelines, validated risk of bias assessment tools (JBI, RoB 2, and Newcastle-Ottawa Scale), and predefined eligibility criteria, will enhance transparency and reproducibility.

Additionally, the planned meta-analysis, where feasible, will allow for quantitative synthesis of effect estimates, thereby increasing statistical power and enabling exploration of between-study heterogeneity through subgroup and sensitivity analyses. Stratification based on study design, population characteristics, and outcome measures is expected to provide nuanced insights into the observed associations.

### Potential Limitations

Several limitations are anticipated. First, heterogeneity in study designs, outcome definitions, and measurement tools may limit the feasibility of meta-analysis for certain outcomes. Second, the inclusion of observational studies may introduce residual confounding, despite efforts to assess and report risk of bias systematically. Third, variability in reporting standards and incomplete adjustment for key confounders, such as physical activity levels, obesity, duration of diabetes, and medication use, may affect the strength of pooled estimates.

Publication bias is another potential concern, particularly if smaller studies with null findings remain unpublished. Where sufficient studies are available, funnel plot analysis and statistical tests for small-study effects will be conducted to address this issue.

### Implications for Future Research

This review aims to pinpoint methodological gaps in the existing body of evidence, such as the necessity for standardized evaluation protocols, extended follow-up periods, and thorough control of confounding variables. The results might also highlight the significance of integrating multidimensional cardiopulmonary evaluations in upcoming diabetes studies.

Future studies should prioritize prospective designs and intervention-based approaches to determine causal relationships and evaluate whether improvements in CRF translate into meaningful clinical benefits for individuals with T2DM.

### Conclusions

This protocol for a systematic review and meta-analysis details a comprehensive method for compiling evidence on CRF in individuals with T2DM. By thoroughly assessing and combining existing data across 4 primary outcome areas—PFT parameters (FEV1 and FVC), 6MWT distance, HRV indexes, and VO₂ max—this review seeks to offer clinically significant, quantitative insights into the degree and factors of cardiorespiratory impairment in T2DM. The results are anticipated to inform evidence-based clinical assessment strategies and direct future research on integrated cardiopulmonary and autonomic evaluation in diabetes care.

## Abbreviations

6MWT: 6-minute walk test
CRF: cardiorespiratory fitness
FEV1: forced expiratory volume in 1 second
FVC: forced vital capacity
GRADE: Grading of Recommendations Assessment, Development and Evaluation
HbA1c: hemoglobin A1c
HRV: heart rate variability
MD: mean difference
PFT: pulmonary function test
PRISMA: Preferred Reporting Items for Systematic Reviews and Meta-Analyses
PRISMA-P: Preferred Reporting Items for Systematic Review and Meta-Analyses Protocols
RoB 2: Revised Cochrane Risk-of-Bias Tool for Randomized Trials
SMD: standardized mean difference
T2DM: type 2 diabetes mellitus
VO2 max: maximal oxygen uptake

## Data Availability

Data sharing is not applicable to this article as no data sets were generated or analyzed during this study.
